# Preoperative optimization of patient expectations improves long-term outcome in heart surgery patients: results of the randomized controlled PSY-HEART trial

**DOI:** 10.1186/s12916-016-0767-3

**Published:** 2017-01-10

**Authors:** Winfried Rief, Meike C. Shedden-Mora, Johannes A. C. Laferton, Charlotte Auer, Keith J. Petrie, Stefan Salzmann, Manfred Schedlowski, Rainer Moosdorf

**Affiliations:** 1 Division of Clinical Psychology, University of Marburg, Gutenbergstrasse 18, Marburg, 35032 Germany; 2 Department of Psychosomatic Medicine and Psychotherapy, University Medical Center Hamburg Eppendorf, Hamburg, Germany; 3 Department of Psychological Medicine, University of Auckland, Auckland, New Zealand; 4 Institute of Medical Psychology and Behavioral Immunobiology, University Hospital Essen, Essen, Germany; 5 Department of Cardiac and Thoracic Vessel Surgery, Heart Centre, University of Marburg, Marburg, Germany

## Abstract

**Background:**

Placebo effects contribute substantially to outcome in most fields of medicine. While clinical trials typically try to control or minimize these effects, the potential of placebo mechanisms to improve outcome is rarely used. Patient expectations about treatment efficacy and outcome are major mechanisms that contribute to these placebo effects. We aimed to optimize these expectations to improve outcome in patients undergoing coronary artery bypass graft (CABG) surgery.

**Methods:**

In a prospective three-arm randomized clinical trial with a 6 month follow-up, 124 patients scheduled for CABG surgery were randomized to either a brief psychological pre-surgery intervention to optimize outcome expectations (EXPECT); or a psychological control intervention focusing on emotional support and general advice, but not on expectations (SUPPORT); or to standard medical care (SMC). Interventions were kept brief to be feasible with a heart surgery environment; “dose” of therapy was identical for both pre-surgery interventions. Primary outcome was disability 6 months after surgery. Secondary outcomes comprised further clinical and immunological variables.

**Results:**

Patients in the EXPECT group showed significantly larger improvements in disability (−12.6; −17.6 to −7.5) than the SMC group (−1.9; −6.6 to +2.7); patients in the SUPPORT group (−6.7; −11.8 to 1.7) did not differ from the SMC group. Comparing follow-up scores and controlling for baseline scores of EXPECT versus SUPPORT on the variable disability only revealed a trend in favor of the EXPECT group (*P* = 0.09). Specific advantages for EXPECT compared to SUPPORT were found for mental quality of life and fitness for work (hours per week). Both psychological pre-surgery interventions induced less pronounced increases in pro-inflammatory cytokine concentrations reflected by decreased interleukin-8 levels post-surgery compared to changes in SMC patients and lower interleukin-6 levels in patients of the EXPECT group at follow-up. Both pre-surgery interventions were characterized by great patient acceptability and no adverse effects were attributed to them. Considering the innovative nature of this approach, replication in larger, multicenter trials is needed.

**Conclusions:**

Optimizing patients’ expectations pre-surgery helps to improve outcome 6 months after treatment. This implies that making use of placebo mechanisms has the potential to improve long-term outcome of highly invasive medical interventions. Further studies are warranted to generalize this approach to other fields of medicine.

**Trial registration:**

Ethical approval for the study was obtained from the IRB of the Medical School, University of Marburg, and the trial was registered at (NCT01407055) on July 25, 2011.

**Electronic supplementary material:**

The online version of this article (doi:10.1186/s12916-016-0767-3) contains supplementary material, which is available to authorized users.

## Background

Placebo mechanisms contribute substantially to clinical treatments in various fields of medicine, but systematic approaches to utilize these mechanisms for improved outcome are scarce [1, 2]. While placebo effects are substantial for patient-reported outcomes such as pain or depression, placebo effects can be also demonstrated for objective parameters such as immune responses, cardiovascular parameters, dopamine release, electroencephalogram and functional magnetic resonance imaging parameters [3]. Major determinants of placebo mechanisms are patient pre-treatment expectations about treatment effects; experimental manipulations of volunteer expectations can either amplify or abolish the pain-reducing effects of potent opioids such as remifentanil [4]. Labeling of treatments substantially determines treatment effects [5]. Therefore, optimizing patient expectations could offer options to improve treatment outcome.

Patient expectations are also associated with favorable outcome of surgical interventions [6–8]. If patients undergoing coronary artery bypass graft surgery (CABG) expected to remain disabled after the surgery, it is more likely that these patients would still suffer from substantial disability post-surgery, even if their surgeons predicted a good patient recovery [9–11]. However, such patient expectations are naturally occurring expectations, and not systematically induced expectations during clinical encounters. Considering the close association between pre-treatment expectations and disability following surgery, the question arises whether optimizing patient preoperative expectations can improve outcome following highly invasive interventions such as CABG. Here, we report on the long-term effects (6 months) of a randomized controlled trial investigating PSYchological preoperative interventions to improve outcome in HEART surgery patients (PSY-HEART trial).

In cardiac surgery, psychological preoperative interventions have been shown to change general risk factors and cardiac misconceptions, to improve knowledge about their surgery, and to increase physical activity [12–14]. However, the results of current preoperative interventions on outcome variables in CABG remain inconclusive [15]. Notably, none of these preoperative interventions directly targeted patient expectations as the most prominent placebo mechanism.

The integration of psychological preparations into the cardiac surgery unit environment requires a brief format. Therefore, we developed a short psychological pre-surgery intervention to optimize patient outcome expectations. We hypothesized that optimizing patient expectations improves outcome in CABG patients, especially in terms of disability as the primary outcome, but also in terms of general quality of life, subjective fitness for work, physical activity levels, and emotional outcomes. As a potential biological marker of the recovery process, we also assessed immune parameters. To evaluate the specificity of such an intervention, we included another psychological comparison condition offering emotional support and behavioral advice, with a similar “dose” as the expectation group. Both interventions were compared to standard medical care (SMC).

## Methods

### Study design

**Fig. 1:**
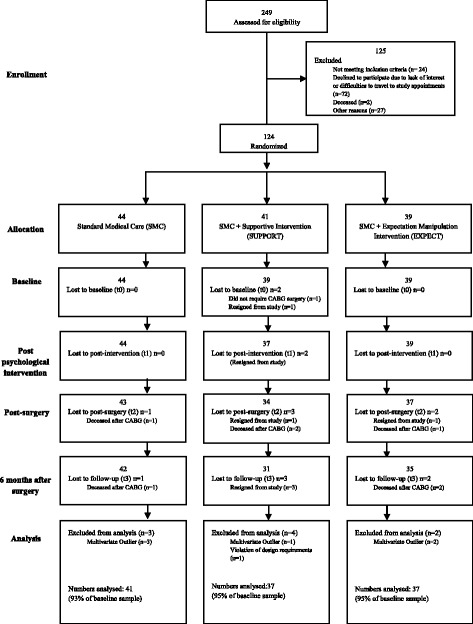
Flow chart (CONSORT). Criteria mentioned in the “Baseline” row and “Analysis” row were reasons for exclusion of the patient from data analyses

This is a longitudinal three arm, randomized clinical trial, investigating the effect of different pre-surgery interventions on 6 months follow-up assessments in patients undergoing heart surgery (see CONSORT flow chart Fig. 1; full description of study design see [16]). We hypothesized that optimizing patient outcome expectations improves long-term outcome even after highly invasive interventions such as heart surgery. After study inclusion, patients were either randomized to an expectation optimization group (EXPECT), or to an emotional support group (SUPPORT), or to SMC preparation for the surgery (for short descriptions of interventions, see below). Clinical outcomes are compared between baseline and 6 months follow-up; immune parameters are also reported for direct surgery-associated changes (see below).

### Participant enrollment

The study took place at the Department of Cardiovascular Surgery, Heart Center, in collaboration with the Division of Clinical Psychology, Philipps University Marburg. Patients on the waiting list of the Heart Surgery Center were contacted before hospital admission. Inclusion criteria were adults older than 18 years who were scheduled for elective on pump CABG or CABG combined with valve surgery. Further inclusion criteria were ability to give informed consent and sufficient fluency in German. The interventions were introduced as two additional, slightly different brief psychological interventions, both aimed at improving coping with CABG. Exclusion criteria were the presence of a serious comorbid non-cardiac medical condition or psychiatric condition that substantially affected disability. Current psychiatric condition was assessed with the standardized interview structured clinical interview for DSM-IV diagnoses [17]. All participants gave written informed consent. Data collection lasted from April 2011 to May 2015.

Out of 249 patients approached for participation, 72 (28.9%) declined because of motivational reasons, including travel problems to attend the study appointments. Patients who agreed to take part in the study were significantly younger (t(157) = 3.31; *P* = 0.001), while sex ratios were comparable to patients who declined [18]. Two patients died before admission to the hospital, while 24 patients did not fulfill the inclusion criteria (Fig. 1). Thus, we started with an ITT sample of 124 patients (87% only CABG; 13% CABG plus heart valve replacement). Follow-up assessments were completed by 108 patients at 6 months follow-up (88.5% of baseline sample; 87% of ITT sample). Seven patients died post-surgery (two in SMC, two in SUPPORT, three in EXPECT condition).

While the study design was not changed from protocol, sample size calculation had to be adapted from initial calculations due to slower than anticipated recruitment into the trial. The sample size was adjusted to 124, thereby ensuring that this is still able to detect at least moderate effects (Cohen’s d > 0.30; alpha = 0.05; clinically meaningful difference in pain disability index > 4) with a power of 85%. Considering the Helsinki recommendation that trials investigating innovative interventions should not be oversized, this was considered acceptable.

### Outcome variables

The predefined primary outcome variable according to the study protocol [16] was disability 6 months after surgery. We used a modified version of the Pain Disability Index, which was adapted for cardiology patients. This scale assesses disability in seven areas of life (family, job, social activities, etc.; ratings from 0 to 10) caused by the major health problem. It offers the opportunity to compare ratings with general population data [19], and results in a disability total score.

Secondary outcome variables addressed quality of life (QoL), fitness for work, physical activity, cardiac anxiety, and mental health. Health-related QoL was assessed by the Short Form Health Survey which has two subscales of QoL, namely physical and mental QoL [20]. Fitness for work was assessed asking patients the amount of time they feel able to work per week (in hours). We also assessed physical activity with the International Physical Activity Questionnaire (IPAQ), which allows the computation of metabolic equivalents of physical exercise [21]. Depression and anxiety were assessed by Hospital Anxiety and Depression Scale [22]. We also assessed cardiac specific anxiety using the Cardiac Anxiety Questionnaire [23]. This scale asks for concerns associated with the experience of cardiac sensations (e.g., after palpitations). Medical outcome variables such as readmission rates, adverse cardiac events after CABG, and acceptability of psychological interventions were also evaluated.

As a manipulation check measure, patient expectations about outcome and personal control beliefs were assessed using subscales of the Expected Illness Perception Questionnaire, which is based upon the Illness Perception Questionnaire [24]. This scale assesses patient expectations about their illness 6 months after surgery. Outcome expectations were assessed by the three items from the “treatment control subscale”, such as “6 months after CABG surgery, the surgery has cured my coronary disease”. Expected personal control beliefs were assessed by the four items from the Expected Illness Perception Questionnaire “personal control subscale”, such as “6 months after CABG surgery, there is a lot I can do myself to control my symptoms”.

As biological markers of the recovery process, immune parameters (interleukin 6 and 8 (IL-6, IL-8), tumor necrosis factor TNF-alpha, C-reactive protein (CRP)) were analyzed from blood samples. They were obtained at baseline, pre-surgery, 6–8 days post-surgery, and at follow-up, standardized at 2:00 PM to control for diurnal variations. Plasma for CRP and cytokine measurements was separated by centrifugation and stored at −80 °C until analysis. Plasma levels were analyzed by flow cytometry using bead-based assays (Bio-Plex Pro Human Cytokine Assays, Bio-Rad Laboratories, Hercules, CA, USA).

### Medical status

Medical status was either assessed directly by physicians of the university hospital, or gathered from the patients’ medical records. It included the New York Heart Association Classification, EuroSCORE II (European System for Cardiac Operative Risk Evaluation [25]), left ventricular ejection fraction (LVEF), comorbid medical conditions, body mass index, smoking status, and history of myocardial infarction.

### Procedure

The first assessment took place 1 week before surgery, either at home or in the university department. This was followed by the first in-person session of the psychological intervention, two phone calls, admission to the hospital, and second in-person session with subsequent assessments of psychological variables, on the day before surgery. Follow-up assessments took place 6 months after surgery. Further characteristics of the study sample can be found in Table 1. Further details of the study design are reported elsewhere [16].

**Table 1:** Demographical, medical and psychological characteristics at baseline of patients receiving Standard Medical Care (SMC; 44), Supportive Intervention (SUPPORT; 39) or Expectation Manipulation Intervention (EXPECT; 39)

	SMC	SUPPORT	EXPECT	Test statistic
Age in years, median (SD)	67.07 (8.9)	64.62 (8.1)	65.76 (7.8)	*F*(2, 112) = 0.856; *P* = 0.427
Sex, male, n (%)	36 (87.8)	30 (81)	32 (86.5)	*χ* ^2^ (2) = 0.768; *P* = 0.681
Education, high school, n (%) (MD = 1)	7 (17.1)	10 (27)	10 (27)	*χ* ^2^ (2) = 1.304; *P* = 0.521
Marital status, married, n (%) (MD = 1)	33 (80.5)	34 (91.9)	31 (83.8)	*χ* ^2^ (2) = 2.097; *P* = 0.350
BMI, median (SD) (MD = 3)	29.67 (5.2)	29.5 (6.6)	29.03 (5.01)	*F*(2, 109) = 0.13; *P* = 0.876
Smoking status, n (%)	6 (14.6)	2 (5.4)	6 (16.2)	*χ* ^2^ (2) = 2.383; *P* = 0.304
EuroSCORE II, median (SD) (MD = 11)^a^	1.53 (0.8)	1.47 (0.8)	1.25 (0.8)	*F*(2, 101) = 2.30; *P* = 0.105
NYHA, n (%) (MD = 10)				*χ* ^2^ (6) = 4.644; *P* = 0.590
I	1 (2.4)	1 (2.7)	0 (0)	
II	9 (22.0)	11 (29.7)	12 (32.4)	
III	28 (68.3)	20 (54.1)	17 (45.9)	
IV	1 (2.4)	2 (5.4)	3 (8.1)	
LVEF, n (%) (MD = 9)				*χ* ^*2*^ (4) = 9.944; *P* = 0.041
≥ 50	23 (48.8)	19 (51.4)	30 (78.4)	
49–30	13 (31.7)	13 (35.1)	4 (10.8)	
< 30	2 (4.9)	2 (5.4)	0 (0)	
Previous myocardial infarction, n (%) (MD = 5)	9 (23.1)	6 (17.1)	6 (16.7)	*χ* ^*2*^ (4) = 2.398; *P* = 0.663
Combined surgery, n (%)	6 (14.6)	6 (16.2)	3 (8.1)	*χ* ^*2*^ (2) = 1.214; *P* = 0.545
Current mental disorder, n (%) (MD = 1)	4 (9.8)	5 (13.5)	8 (21.6)	*χ* ^2^ (2) = 2.130; *P* = 0.345
Anxiety, median (SD) (MD = 7)^b^	4.03 (3.0)	4.55 (3.2)	5.17 (5.0)	*F*(*2*, 105) = 0.838; *P* = 0.435
Depression, median (SD) (MD = 8)^c^	4.59 (3.1)	4.0 (3.1)	5.11 (4.0)	*F*(*2*, 104) = 0.894; *P* = 0.412
Disability, median (SD) (MD = 3)^d^	22.78 (13.8)	22.14 (15.3)	25.89 (15.0)	*F*(2, 110) = 0.683; *P* = 0.507
Mental quality of life, median (SD) (MD = 7)^e^	48.96 (9.9)	51.6 (8.7)	47.98 (12.8)	*F*(2, 105) = 1.081; *P* = 0.343
Physical quality of life, median (SD) (MD = 5)^f^	40.26 (10.5)	39.55 (11.0)	37.15 (9.2)	*F*(2, 107) = 0.920; *P* = 0.402
^g^Physical activity, median (SD) (MD = 20)	2668.4 (2146.2)	2635.5 (2806)	2433.6 (3339.5)	*F*(2, 92) = 0.067; *P* = 0.935
^h^Cardiac anxiety, median (SD) (MD = 4)	2.48 (0.6)	2.68 (0.6)	2.67 (0.7)	*F*(2, 108) = 1.260; *P* = 0.288

^a^ *EuroSCORE* European System for Cardiac Operative Risk Evaluation; untransformed data is displayed to facilitate interpretation; statistical analyses are based on log-transformed data

^b^Anxiety (Hamilton Anxiety and Depression Scale; HADS) range = 0–21

^c^Depression (HADS) range = 0–21

^d^Disability (Pain Disability Index) range = 0–70

^e^Mental quality of life (Mental component of the Short-Form Health Survey (SF-12))

^f^Physical quality of life (Physical component of the SF-12)

^g^Physical activity (International Physical Activity Questionnaire, weighted estimate of total physical activity per week

^h^Cardiac Anxiety Questionnaire, range = 0–4

*SMC* Standard Medical Care, *SUPPORT* Supportive Intervention, *EXPECT* Expectation Manipulation Intervention, *BMI* body mass index, *NYHA* New York Heart Association functional classification, *LVEF* left ventricular ejection fraction, *MD* missing data

Assignment to treatment arms followed a stratified permuted block randomization procedure with a block size of 9. Stratification criteria were age (above or below 65 years) and New York Heart Association class (1,2 versus 3,4) to control for differences in cardiac status. Random procedure was defined using an internet program (WINPEPI) before first-patient-in by JL, enrollment of patients was initiated by a study nurse being blind with regards to treatment condition. Allocation concealment was verified using closed envelopes including group allocation information that were handed over to the therapist after inclusion of a new patient. Surgeons, hospital staff involved in patient care, and staff assessing treatment effects were blind to treatment condition.

### Interventions

EXPECT and SUPPORT both encompassed the same amount of personal contact (two 50 min individual sessions pre-surgery, two 20 min phone calls pre-surgery, one 20 min booster phone call post-surgery). Treatment sessions were audiotaped to verify treatment adherence. The brief and focused format of the interventions was shown to be feasible in the cardiac surgery environment.

EXPECT (expectation manipulation intervention): This intervention focused on the development of realistic expectations about the benefits of surgery and the recovery process. Patients were encouraged to develop personal ideas and images about their future after surgery, including plans about activities and how they will enjoy their life afterwards (outcome expectations). Personally relevant steps and plans for the 6 months after surgery were recorded for patients. Additionally, patients received a booklet containing all relevant session information, including the work sheets and audio-CDs of their sessions. Finally, normal symptoms after surgery that could be expected were discussed, and differentiated from unlikely complications. Patient control expectations were enhanced by discussing ways in which they could manage unpleasant symptoms or sensations, and how they could positively influence the disease course after surgery.

An example may further illustrate this intervention. Many patients hoped to again be able to work in their garden after surgery. In the EXPECT intervention, these patients developed specific plans on how they would successfully be able to reassume gardening activities due to their expected increased exercise capacity following surgery: repotting small plants in the early stage, lawn mowing after some time, increasing to more demanding gardening tasks between 3–6 months after surgery. One patient imagined himself chopping wood in preparation for hosting a barbecue in his garden for his family.

SUPPORT: This attention control group received the same amount of therapist attention, but without targeting expectations. Therapists encouraged expressing emotions and anxieties about the anticipated surgery, and therapists used reflective listening techniques and expressed empathy. This therapy has been developed to include all so-called “common factors of psychotherapy”, such as empathy, therapist attention, and verbalization of emotions [26]. Patients in the SUPPORT group did not receive audio-CDs.

SMC: Like the patients of the other groups, these patients received the standardized informed consent procedure before surgery, and general medical care, but no additional psychological interventions. Assessments were identical.

Therapists: Pre-surgery interventions were provided by three psychologists (2 male, 1 female). All therapists were specifically trained and provided both types of interventions; they were additionally supervised by a senior psychotherapist to ensure treatment fidelity. Previous analyses confirmed treatment fidelity, and treatment satisfaction was similar between the two intervention groups [18].

### Statistics

The primary hypotheses (better follow-up outcome in the EXPECT group) were analyzed using a linear mixed model with time, treatment group and time × treatment group interaction as fixed effects and a random intercept for subject specific effects with maximum likelihood estimation and autoregressive residual matrix. Compared to intention-to-treat analyses, this procedure provides better estimates for missing data using the full data sample (intention-to-treat sample), and addresses individual differences more adequately [27, 28]. Pattern of missing values is postulated to be random. We expected a significant interaction between intervention group and assessment time. Requirements of this procedure for data distribution were inspected according the Field’s recommendations [29]. If criteria for multivariate outliers were fulfilled (1 to 3 persons per group; Mahalanobis-distance criteria), preconditions for maximum likelihood estimations were violated, and data were not included. If significant interactions occurred, we report pre–post tests per group to indicate whether the specific group has improved, and we compared follow-up scores of pairwise groups controlling for baseline scores (two groups, two assessment points).

For immunological parameters, preconditions for multilinear analyses were checked, and log-transformation was used if data was extremely skewed and could not be used for calculating linear mixed models (this was the case for IL-6). Boxplots were used to check for outliers. Values greater/lower than three interquartile ranges from the upper/lower quartile were considered as missing values. This was the case for less than 5.4%.

All statistical analyses were run using SPSS Statistics 22. Tables report observed means for all variables, figures report estimated marginal means for selected variables to illustrate effects.

## Results

### Baseline characteristics

Despite large comparability of baseline variables (Table 1), we found differences for LVEF, with more favorable scores in the EXPECT group. While we continued to analyze the data as planned, we also repeated the central statistical analyses out-of-protocol adjusting for LVEF as covariate [30]; however, significant findings of group × assessment point, e.g., for disability, QoL, and physical activity were replicated.

### Manipulation check

**Fig. 2:**
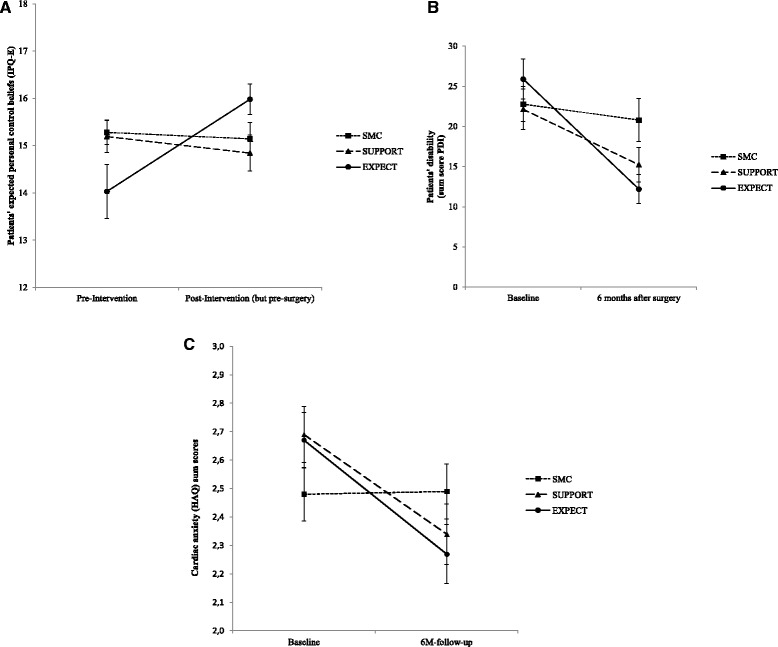
Manipulation check (expectations pre- versus post-psychological intervention). Patients’ expected personal control (**a**), patients’ disability (**b**) and cardiac anxiety (**c**) improvements from baseline to 6 months follow-up. *SMC* Standard Medical Care, *SUPPORT* Supportive Therapy, *EXPECT* Expectation Manipulation Intervention. Data from estimated marginal means analyses

Specific effects of our expectation manipulation on patient beliefs about their ability to have some control over the course of the illness and recovery were confirmed by a significant interaction between time × treatment group. Patient expectations to personally control the disease were significantly higher after the psychological intervention (simple effects per group compared to baseline) for EXPECT (from 14.03 to 16.06; *P* < 0.001), but not for SUPPORT (from 15.24 to 14.91; *P* = 0.409) or SMC (from 15.28 to 15.16, *P* = 0.743; Fig. 2a).

### Primary outcome: disability at 6 months

For our primary outcome disability, a better outcome in the EXPECT group was found, indicated by a significant group × time interaction. Improvements in disability were significantly larger in the EXPECT (−12.6; −17.6 to −7.5) compared to the SMC group (−1.9; −6.6 to +2.7), with intermediate effects in the SUPPORT group (−6.7; −11.8 to 1.7). The decrease in disability between baseline and follow-up was significant in the EXPECT (simple time effects per group *P* < 0.001) and in the SUPPORT group (*P* = 0.01), but not in the SMC group (*P* = 0.404) (Fig. 2b). Further post hoc tests used two-group comparisons, controlling for corresponding baseline scores (two groups, two assessment points). The significant advantage of EXPECT over SMC was confirmed (interaction with two groups as post hoc test; F(1, 70.442) = 9.562, *P* = 0.003), while the SUPPORT group did not report significantly lower disability scores than the SMC group (F(1, 71.578) = 1.781, *P* = 0.186). Comparing the two groups with psychological preoperative interventions, we found a trend in favor of the EXPECT group compared to the SUPPORT group (interaction of the two groups with assessment time F(1, 62.571) = 2.872, *P* = 0.095), which failed to reach significance. Additional file 1: Figure S4 shows individual courses of improvement in disability scores between the three intervention groups.

To test a possible mediation of expectation changes for our primary outcome, we repeated the main analysis on disability, once with personal control expectations at baseline as covariate, second with personal control expectations after the psychological interventions as covariate. Adding the baseline variable “control expectation” as a covariate further sharpens the significant group × time interaction for disability (F = 5.4; *P* = 0.006), without showing any significant effect for the covariate (F = 0.15; ns). Including “control expectation after the psychological interventions” shows more potential as a mediator and leads to the non-significance of the group × time interaction (F = 2.14; *P* = 0.12), but the covariate still fails to contribute significantly (F = 2.08; *P* = 0.15).

### Secondary outcomes

The better outcome for the EXPECT group was further confirmed by QoL data as assessed by the Short-Form Health Survey. For mental QoL, a significant time × treatment group interaction indicated that mental QoL increased for patients in the EXPECT group at follow-up compared to baseline (*P* < 0.001), but not for patients’ receiving SUPPORT (*P* = 0.748) or SMC (*P* = 0.329). Post hoc tests revealed significant advantages for the EXPECT group compared to SMC (F = 5.9; *P* = 0.018), but also compared to SUPPORT (F = 7.3; *P* = 0.009), while the SUPPORT group was similar to SMC (F = 0.2; non-significant). For physical QoL, both psychological intervention groups reported better outcome than the SMC group, indicated by an overall group × assessment interaction, and significant improvements in both psychological intervention groups (Table 2). Post hoc tests revealed significant advantages of EXPECT versus SMC (F = 6.3; *P* = 0.015), while the other comparisons of two groups were non-significant (SUPPORT vs. SMC F = 3.1, *P* = 0.081; EXPECT vs. SUPPORT F = 0.3, non-significant).

**Table 2:** Outcome measures at baseline and 6 months after surgery of patients receiving standard medical care (SMC), Supportive Intervention (SUPPORT) or Expectation Manipulation Intervention (EXPECT) (observed means)

	(A) SMC	(B) SUPPORT	(C) EXPECT	Test statistic (F scores indicate interaction terms)	Significant pre–post comparisons
Disability (PDI)					
Baseline	22.78 (18.36–27.19)	22.14 (17.04–27.23)	25.89 (20.81–30.97)	*F*(2, 102.542) = 4.762;	B, C
Follow-up	20.79 (15.35–26.23)	15.23 (10.82–19.65)	12.19 (8.50–15.89)	*P* = 0.011	
Mental quality of life (SF-12)					
Baseline	48.96 (45.71–52.20)	51.60 (48.56–54.63)	47.98 (43.66–52.31)	*F*(2, 94.803) = 4.627;	C
Follow-up	50.58 (47.02–54.14)	52.40 (48.83–55.97)	56.74 (54.29–59.19)	*P* = 0.012	
Physical quality of life (SF-12)					
Baseline	40.26 (36.80–43.73)	39.55 (35.82–43.29)	37.15 (34.04–40.26)	*F*(2, 103.928) = 3.186;	B, C
Follow-up	40.28 (36.58–43.98)	45.21 (41.23–49.19)	44.77 (41.13–48.41)	*P* = 0.045	
Patient subjective working ability in hours per week follow-up	15.97 (10.50–21.43)	17.14 (10.45–23.80)	25.40 (19.90–30.90)	*F(*2, 74) = 3.325; *P* = 0.041^a^	AC^a^, BC^a^
Physical activity levels (IPAQ)					
Baseline	2668.40 (1919.56–3417.24)	2635.54 (1547.50–3723.57)	2433.63 (1249.51–3617.75)	*F*(2, 72.915) = 4.776;	B, C
Follow-up	2957.64 (2128.50–3786.78)	5388.21 (3618.71–7157.71)	4700.60 (3470.76–5930.45)	*P* = 0.011	
Cardiac anxiety (CAQ)					
Baseline (pre-intervention)	2.48 (2.29–2.67)	2.68 (2.47–2.90)	2.67 (2.44–2.89)	F(2, 107.021) = 3.980;	B, C
Follow-up (after 6 months)	2.50 (2.31–2.69)	2.34 (2.11–2.57)	2.29 (2.11–2.47)	*P* = 0.022	
Anxiety (HADS)					
Baseline (pre-intervention)	4.03 (3.07–4.98)	4.55 (3.42–5.67)	5.17 (3.47–6.86)	F(2,102.409) = 1.325;	
Follow-up (after 6 months)	3.24 (2.42–4.05)	2.52 (1.52–3.51)	3.61 (2.22–4.99)	*P* = 0.270	
Depression (HADS)					
Baseline (pre-intervention)	4.59 (3.60–5.58)	4.00 (2.89–5.11)	5.11 (3.75–6.47)	F(2, 99.211) = 2.604;	
Follow-up (after 6 months)	3.68 (2.62–4.75)	1.88 (1.20–2.57)	2.58 (1.60–3.56)	*P* = 0.079	
Outcome expectations (IPQ-E)					
Baseline (pre-intervention)	12.05 (11.49–12.62)	12.61 (12.10–13.12)	12.37 (11.90–12.83)	F(2, 98.882) = 0.084; *P* = 0.919	
Post psychological intervention	11.94 (11.32–12.56)	12.29 (11.97–12.60)	12.09 (11.52–12.66)		
Expected personal control (IPQ-E)					
Baseline (pre-intervention)	15.28 (14.74–15.81)	15.24 (14.55–15.92)	14.03 (12.90–15.16)	F(2, 103.971) = 8.748; *P* < 0.001	C
Post psychological intervention	15.16 (14.43–15.88)	14.91 (14.13–15.68)	16.06 (15.40–16.73)		
Patients rehospitalized after CABG, n (%)^a^					
Follow-up	9 (26.47)	7 (23.33)	3 (9.38)	*χ* ^2^ (2) = 3.380; *P* = 0.185	
LVEF, n (%) at follow-up					
≥ 50	25 (80.6)	23 (88.5)	24 (100)	*χ* ^*2*^ (2) = 5.138; *P* = 0.077	
49–30	6 (19.4)	3 (11.5)	0 (0)		
< 30	0 (0)	0 (0)	0 (0)		
Interleukin-6 (pg/mL)^a^					
Baseline (pre-intervention)	3.71 (2.14–5.27)	2.10 (1.34–2.86)	3.58 (1.91–5.25)	F = (6, 216.924) =2.523; *P* = 0.022	Follow-up: AC
Post psychological intervention	3.06 (2.03–4.10)	1.83 (1.44–2.22)	3.44 (2.07–4.82)		
After surgery	24.75 (18.61–30.89)	19.56 (13.63–25.49)	19.90 (15.63–24.18)		
Follow-up (after 6 months)	4.83 (2.99–6.68)	3.66 (2.16–5.16)	2.39 (1.38–3.41)		
Interleukin-8 (pg/mL)					
Baseline (pre-intervention)	5.48 (4.05–6.90)	5.56 (4.33–6.79)	5.78 (4.18–7.38)	F = (6, 206.632) = 4.186; *P* = 0.001	Post-surgery: AB, AC
Post psychological intervention	4.76 (3.86–5.66)	5.13 (3.89–6.38)	5.52 (3.73–7.30)		
After surgery	13.71 (11.44–15.97)	10.31 (8.14–12.48)	11.02 (8.64–13.40)		
Follow-up (after 6 months)	5.08 (3.63–6.52)	4.83 (4.22–5.45)	6.07 (4.76–7.37)		
Tumor necrosis factor alpha (pg/mL)^a^					
Baseline (pre-intervention)	2.01 (1.60–2.42)	1.48 (1.19–1.76)	1.61 (1.39–1.83)	F(6, 174.813) = 1.404; *P* = 0.216	
Post psychological intervention	2.20 (1.78–2.62)	1.54 (1.32–1.76)	1.72 (1.43–2.01)		
After surgery	3.63 (2.72–4.54)	2.17 (1.81–2.52)	3.12 (2.51–3.72)		
Follow-up (after 6 months)	2.66 (2.00–3.32)	1.72 (1.48–1.96)	2.09 (1.70–2.49)		
C-reactive protein (μg/mL)^a^					
Baseline (pre-intervention)	2.49 (1.84–3.15)	2.23 (1.32–3.15)	2.96 (2.00–3.92)	F(6, 209.520) = 1.410; *P* = 0.212	
Post psychological intervention	2.22 (1.62–2.81)	2.19 (1.52–2.86)	3.42 (2.07–4.76)		
After surgery	123.93 (94.24–153.62)	101.71 (77.65–125.76)	96.14 (76.91–115.38)		
Follow-up (after 6 months)	4.23 (2.05–6.42)	4.17 (1.96–6.38)	2.33 (1.54–3.12)		

For significant group × time interactions follow-up tests were calculated. ‘Significant pre–post comparisons’ (last column) means that the baseline value was significantly different from the post value for the groups stated in this column (e.g., ‘B, C’ would indicate that for SUPPORT and EXPECT the baseline values were significantly different from the post values within both groups, but for SMC baseline and post values did not differ significantly). For variables only reported at follow-up (e.g., working ability) and for biological variables (four assessment points), the letters indicate significant group differences at the indicated time point. (e.g. ‘Follow-up: AC’ would indicate that groups SMC and EXPECT have significantly different scores at follow-up)

^a^Because this variable was only assessed at follow-up, the column “test statistics” includes group comparison (three groups), while the column “pre–post comparison” includes pairwise comparisons

*PDI* Pain Disability Index, *SF-12* Short-Form Health Survey, *IPAQ* International Physical Activity Questionnaire, *CAQ* Cardiac Anxiety Questionnaire, *HADS* Hamilton Anxiety and Depression Scale, *IPQ-E* Expected Illness Perception Questionnaire, *CABG* coronary artery bypass graft, *LVEF* left ventricular ejection fraction

A significant advantage of the EXPECT group was also found for subjective ability to work at follow-up (Table 2). Only patients of the EXPECT group reported significantly more hours of working ability, compared to SMC. The increase of metabolic equivalents of physical activity after surgery at follow-up was significantly different between groups (IPAQ), with significant increases of physical activity in both intervention groups (EXPECT: *P* <0.001; SUPPORT: *P* < 0.001), but not in the SMC group (*P* = 0.673). Repeated measure analyses of pairs of two groups comparing follow-up scores and controlling for baseline scores confirmed more improvement in the psychological intervention groups versus SMC (EXPECT vs. SMC F = 5.87, *P* = 0.019; SUPPORT vs. SMC F = 10.17, *P* = 0.002), while the two pre-surgery intervention groups did not differ (EXPECT vs. SUPPORT F = 0.14; non-significant).

For cardiac anxiety, a significant interaction (Fig. 2c) indicated that improvements were highly significant in the EXPECT (*P* < 0.001) and the SUPPORT group (*P* < 0.01), but not after SMC. Accordingly, repeated measure analyses of pairs of two groups comparing follow-up scores and controlling for baseline scores confirmed improvements of EXPECT and SUPPORT versus SMC (EXPECT vs. SMC F = 6.78, *P* = 0.011; SUPPORT vs. SMC F = 4.25; *P* = 0.043; EXPECT vs. SUPPORT F = 0.36; non-significant). For depression, significant decreases between admission and follow-up indicated general improvements on this variable, but no group specific changes (main effect for time: F(3, 220.6) = 17.3; *P* < 0.001). A similar pattern was found for general anxiety (main effect for time: F(3, 207.7) = 11.6; *P* < 0.001).

### Immune parameters

**Fig. 3:**
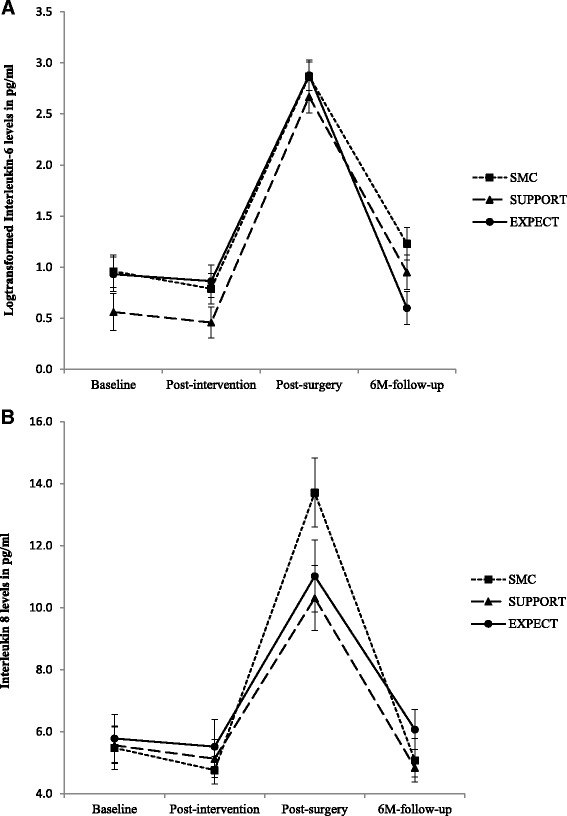
Interleukin-6 (log transformed because of distribution violations) (**a**) and interleukin-8 levels (**b**) by treatment groups at baseline, after intervention, after surgery, and 6 months after surgery. * significant group differences at *P* < 0.05. Data from estimated marginal means analyses. *SMC* standard medical care, *SUPPORT* supportive therapy, *EXPECT* expectation manipulation intervention

A significant change in immune response after surgery was confirmed for pro-inflammatory cytokines and CRP (Table 2; main effects for assessment time IL-6 F(3, 218.319) = 198.192, *P* < 0.001; IL-8 F(3, 208.965) = 93.369, *P* < 0.001; TNF-α F(3, 174.603) = 54.807, *P* < 0.001; CRP F(3, 208.298) = 911.370, *P* < 0.001). Significant interactions between group and assessment time were caused by decreased post-surgery IL-8 levels (Fig. 3b). Pairwise comparisons per assessment point revealed only significant group differences after surgery – both groups with psychological interventions had lower scores than patients in the SMC group (EXPECT *P* = 0.028; SUPPORT *P* = 0.01), with no significant difference between EXPECT and SUPPORT (*P* > 0.20). In addition, patients in the EXPECT group had lower (log-transformed) IL-6 concentrations at follow-up (Fig. 3a). Pairwise comparisons per assessment point revealed one significant finding, namely that EXPECT patients had significantly lower log-IL-6 scores than SMC patients at follow-up (*P* = 0.006).

### Medical outcomes, adverse events

LVEF scores at baseline are reported in Table 1 (*n* = 105), LVEF scores at follow-up are reported in Table 2 (*n* = 81). Most patients achieved satisfactory LVEF scores (>50%) at follow-up, with some advantages in the EXPECT group (100% of patients) compared to the SUPPORT (88.5%) and SMC (80.6%) groups. These differences were significant on a trend level (*χ* ^*2*^ (2) = 5.138; *P* = 0.077); considering cell sizes and baseline differences, this will not be further interpreted. Although rehospitalization scores after surgery were lowest in the EXPECT group (9% vs. 23% in the SUPPORT and 26% in the SMC group), this difference failed to achieve statistical significance (*χ* ^2^ (2) = 3.380; *P* = 0.185; Table 2). The groups did not differ substantially in terms of medication intake or adverse cardiac events after CABG or during follow-up (Additional file 1). Acceptability and satisfaction with the psychological interventions was very high without reporting of any negative effects (details see [18]).

## Discussion

Our general research question was whether placebo mechanisms, such as patient expectations, can be utilized to improve outcome in invasive medical interventions such as CABG. We developed a brief psychological intervention that can be carried out in a cardiac surgery environment, and which focuses on the optimization of patients’ expectations about course and outcome after cardiac surgery. Patients who received the expectation-oriented intervention (EXPECT) reported lower disability and improved QoL 6 months after surgery. This result is further underlined by patient ratings about their fitness for work – patients receiving this intervention reported to be able to work significantly more hours per week than patients receiving SMC or a psychological control intervention (SUPPORT). The fact that recovery was significantly improved in the EXPECT group underpins the relevance of specifically targeting patient expectations beyond employing general therapeutic techniques such as an empathic and supportive relationship.

While the SUPPORT group did not achieve the same positive results as the EXPECT group, their outcome was still better than in the SMC group for some (e.g., physical activity, cardiac anxiety), but not all variables (no significant advantage in disability, working ability, QoL, depression, or anxiety). We introduced the SUPPORT group as an “attention control group” to the EXPECT group. However, empathic interactions and a positive therapeutic relationship are also considered to be effective placebo mechanisms, and they can substantially enhance treatment efficacy, while reducing risks for negative events [31–33]. Therefore, the SUPPORT group covers the placebo mechanism of empathy, while the EXPECT group covers both empathy and expectation modification. Similar improvements in cardiac anxiety between SUPPORT and EXPECT group could indicate that SUPPORT offers significant help in the reduction of pre-surgery anxieties, that can even contribute to biological post-surgery processes (e.g., IL-8). However, the study design characteristics could also contribute to the SUPPORT effects – the use of the same trainers for both treatments reduces error variance due to therapist differences, but could carry with it some risk of contamination effects. Although treatment fidelity checks indicated satisfactory adherence to the different treatment protocols, modest contamination effects could still have occurred.

Increases in CRP and pro-inflammatory cytokine levels confirmed the inflammatory response after surgery. However, cytokine levels were also affected by the intervention post-surgery and during the recovery process. Both psychological preoperative interventions induced lower IL-8 increases after surgery. In addition, IL-6 concentrations were the lowest in the EXPECT group at 6 months follow-up. These cytokines may play a major role in the pathogenesis of coronary artery diseases and their treatments [34], although the functional relevance of these effects has to be further investigated. Of note, treatment-specific factors and placebo mechanisms frequently use similar pathways of action, e.g., opioid pathways in placebo analgesia [35], dopaminergic pathways of placebo effects in Parkinson’s disease [36], or neural plasticity effects of context factors in psychopharmacological treatments [37]. Therefore, an immunological pathway of expectation-based interventions in heart surgery patients as one potential trajectory would parallel the findings from other clinical conditions.

In general, most clinical trials in medicine focus on the so-called specific treatment mechanisms, and on how to optimize them. However, to optimize treatment outcome, it is not enough to only optimize surgery procedures, drug compositions, etc. Treatment regimes should also be designed to optimize patient-specific and contextual factors that also contribute to positive treatment outcomes. These person-specific aspects (such as expectation) and contextual factors interact with treatment-specific factors, and must be taken into consideration for optimized treatment planning.

### Limitations

Despite positive effects on our primary outcome disability, for several clinical variables, only a trend in favor of EXPECT was found, and it is unclear whether larger studies could provide even more convincing results (e.g., on variables such as re-hospitalization or LVEF). Rehospitalization rates, for instance, were too low in our trial to show significant group differences. Moreover, our manipulation check analyses confirmed intervention effects on “control expectations”, while specific effects on outcome expectations remained unclear. Larger, multi-centered trials are therefore required not only to generalize from one study site to healthcare systems in general, but also to investigate further clinical outcome variables. Sensitivity analyses and identification of subgroups who maximally benefit from our interventions should follow. Immune parameters should be only interpreted with caution, and need more sophisticated investigations to better understand their functionality. In general, the nature of this trial is on providing first evidence for an innovative approach, while confirmatory trials should follow.

## Conclusions

To conclude, we were able to show that utilization of placebo mechanisms is helpful to improve outcome even in highly invasive medical interventions. A short-term psychological intervention can be feasibly implemented in a cardiac surgery unit, and participating in this intervention improved long-term outcome after CABG, in particular disability and QoL. Compared to previous studies of psychological interventions in cardiac patients, our intervention trial is characterized by a strong focus on expectations, and a large sample size. A replication of this approach and extension to other clinical interventions is warranted.

## Additional file

Additional file 1: Figure S4. Individual courses of disability scores in the three treatment conditions. **Figure S5**. a: Quality of Life (mental). b: Anxiety. c: Depression. **Table S3**. Means and percentages of patients’ medication at hospital admission for Standard Medical Care (SMC), Supportive Therapy (SUPPORT) or Expectation Manipulation Intervention (EXPECT). **Table S4**. Means and percentages of patients’ medication at hospital discharge for Standard Medical Care (SMC), Supportive Therapy (SUPPORT) or Expectation Manipulation Intervention (EXPECT). **Table S5**. Further outcome variables. (DOC 283 kb)

## Abbreviations

CABG: coronary artery bypass graft surgery
CRP: C-reactive protein
EXPECT: psychological intervention focusing on the modification of patient’s expectations
IL-6: Interleukin 6
IL-8: Interleukin 8
IPAQ: International Physical Activity Questionnaire
LVEF: left ventricular ejection fraction
QoL: quality of life
SMC: standard medical care
SUPPORT: psychological Intervention focusing on empathy and emotions of patients
