# Supplementary material for: Preoperative optimization of patient expectations improves long-term outcome in heart surgery patients: results of the randomized controlled PSY-HEART trial
Source: BMC Med. 2017 Jan 10;15:4. doi: 10.1186/s12916-016-0767-3 (PMC5223324; doi:10.1186/s12916-016-0767-3)
Supplement: Additional file 1: Figure S4. — Individual courses of disability scores in the three treatment conditions. Figure S5. a: Quality of Life (mental). b: Anxiety. c: Depression. Table S3. Means and percentages of patients’ medication at hospital admission for Standard Medical Care (SMC), Supportive Therapy (SUPPORT) or Expectation Manipulation Intervention (EXPECT). Table S4. Means and percentages of patients’ medication at hospital discharge for Standard Medical Care (SMC), Supportive Therapy (SUPPORT) or Expectation Manipulation Intervention (EXPECT). Table S5. Further outcome variables. (DOC 283 kb) [file 12916_2016_767_MOESM1_ESM.doc]

**Additional file 1**

Figure S4: Individual courses of disability scores in the 3 treatment conditions


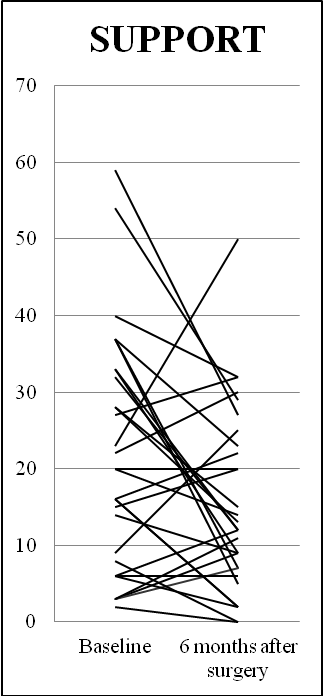

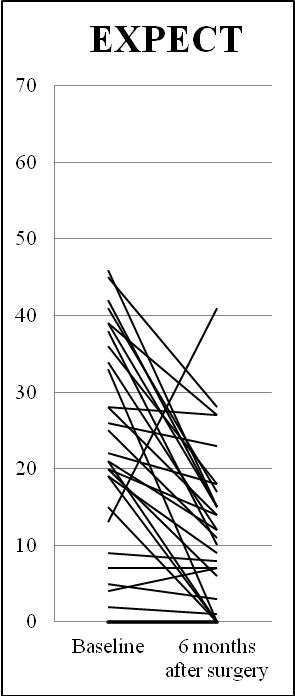

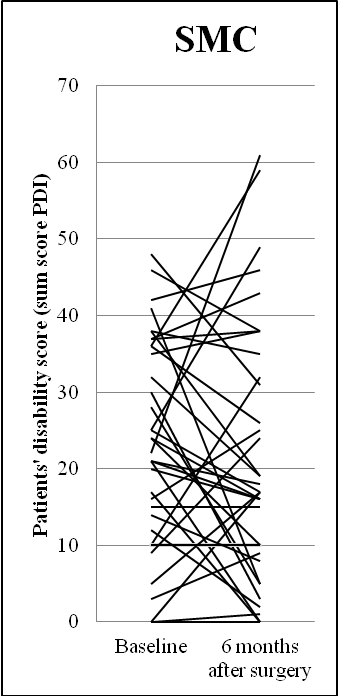
.

**D**

Figure S5a: Quality of Life (mental)


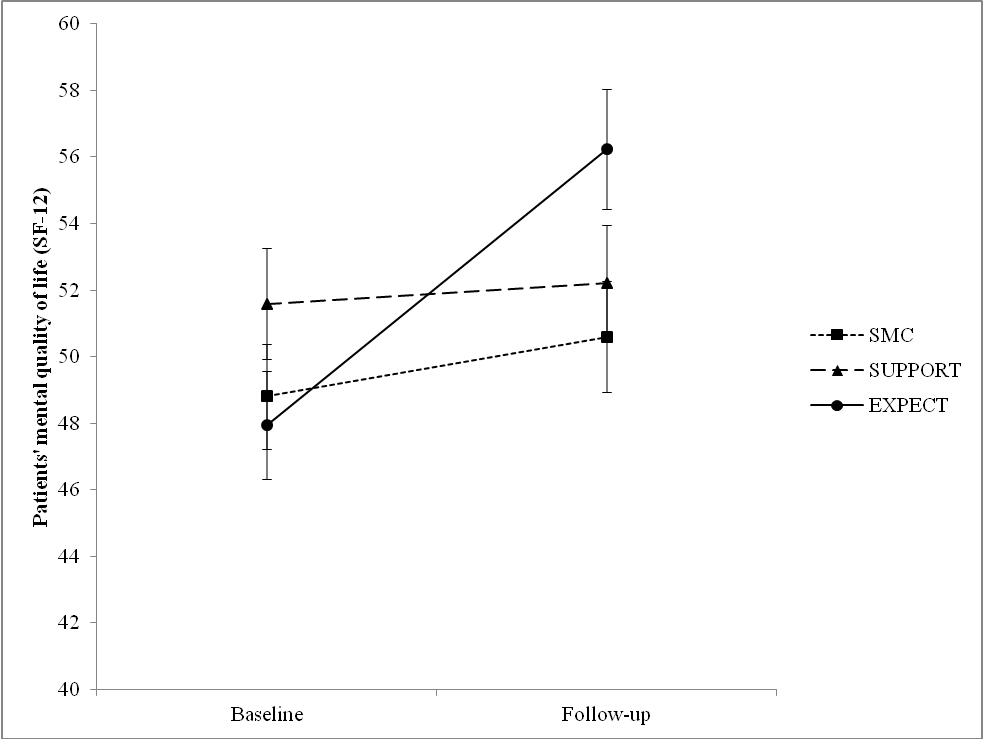


Figure S5b: Anxiety


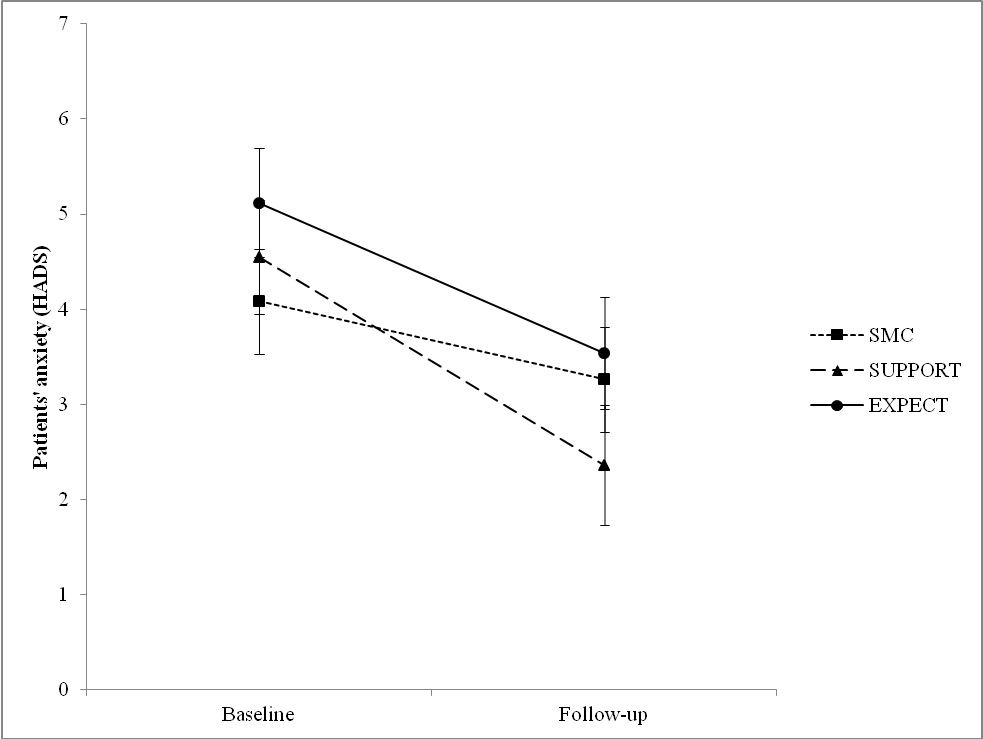


Figure S5c: Depression


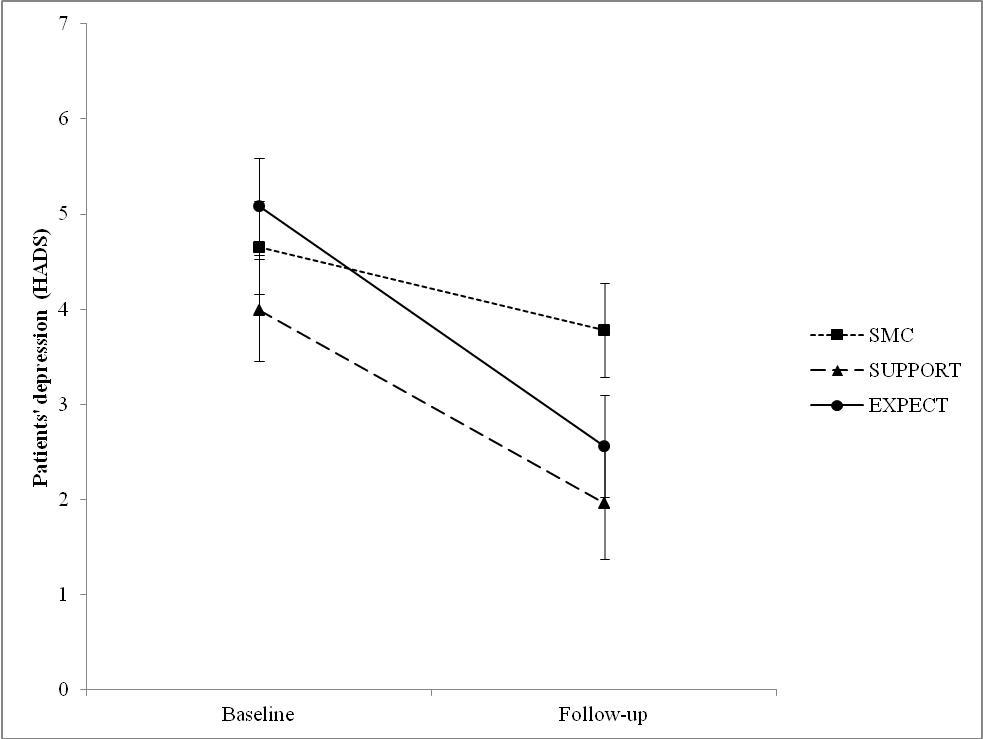


**Table S3. Means and percentages of patients’ medication at hospital admission for Standard Medical Care (SMC), Supportive Therapy (SUPPORT) or Expectation Manipulation Intervention (EXPECT)**

|  | SMC | SUPPORT | EXPECT | test statistic |
| --- | --- | --- | --- | --- |
| Anti-hypertensive drugs *n (%; MD=15)* | 33 (91.7) | 30 (96.8) | 30 (90.9) | *χ*^2^ (2)= 0.998; *P*=.607 |
| Cholesterol-lowering drugs/ statins *n (%; MD=15)* | 17 (47.2) | 17 (54.8) | 13 (39.4) | *χ*^2^ (2)= 1.532; *P*=.465 |
| Analgesic drugs (NSAID^a^) *n (%;MD=15)* | 5 (13.9) | 11 (34.5) | 10 (30.3) | *χ*^2^ (2)= 4.511; *P*=.105 |
| Analgesic opioids *n (%; MD=15)* | 1 (2.8) | 0 (0) | 0 (0) | *χ*^2^ (2)= 1.796; *P*=.407 |
| Other medication^b^ *n (%; MD=15)* | 9 (25.0) | 10 (32.3) | 6 (18.2) | *χ*^2^ (2)= 1.689; *P*=.430 |
| Anti-depressant drugs *n (%; MD=15)* | 2 (5.6) | 2 (6.5) | 0 (0) | *χ*^2^ (2)= 2.087; *P*=.352 |
| Anti-diabetic drugs *n (%; MD=15)* | 4 (11.1) | 6 (19.4) | 2 (6.1) | *χ*^2^ (2)= 2.717; *P*=.257 |
| Antibiotic drugs *n (%; MD=16)* | 0 (0) | 0 (0) | 1 (3.0) | *χ*^2^ (2)= 2.020; *P*=.364 |
| Antiarrhythmic drugs *n (%; MD=15)* | 1 (2.8) | 3 (9.7) | 3 (9.1) | *χ*^2^ (2)= 1.549; *P*=.461 |
| Glucocorticoids *n (%; MD=15)* | 3 (8.3) | 2 (6.5) | 2 (6.1) | *χ*^2^ (2)= 0.157; *P*=.924 |

^a^ Nonsteroidal anti-inflammatory drug (NSAID).

^b^ Anti-depressant, anti-diabetic, anti-biotic, anti-arrhythmic drugs, or glucocorticoids.

**Table S4. Means and percentages of patients’ medication at hospital discharge for Standard Medical Care (SMC**), Supportive Therapy (SUPPORT) or Expectation Manipulation Intervention (EXPECT)

|  | SMC | SUPPORT | EXPECT | test statistic |
| --- | --- | --- | --- | --- |
| Anti-hypertensive drugs *n (%; MD=16)* | 33 (91.7) | 29 (96.7) | 29 (87.9) | *χ*^2^ (2)= 1.639; *P*=.441 |
| Cholesterol-lowering drugs/ statins *n (%; MD=16)* | 17 (47.2) | 14 (46.7) | 15 (45.5) | *χ*^2^ (2)= 0.022; *P*=.989 |
| Analgesic drugs (NSAID^a^) *n (%;MD=16)* | 35 (97.2) | 30 (100.0) | 32 (97.0) | *χ*^2^ (2)= 0.893; *P*=.640 |
| Analgesic opioids *n (%; MD=16)* | 2 (5.6) | 0 (0) | 1 (3.0) | *χ*^2^ (2)= 1.719; *P*=.423 |
| Other medication^b^ *n (%; MD=16)* | 26 (72.2) | 26 (86.7) | 23 (69.7) | *χ*^2^ (2)= 2.849; *P*=.241 |
| Anti-depressant drugs *n (%; MD=17)* | 1 (2.8) | 2 (6.7) | 0 (0.0) | *χ*^2^ (2)= 2.334; *P*=.311 |
| Anti-diabetic drugs *n (%; MD=16)* | 6 (16.7) | 7 (23.3) | 6 (18.2) | *χ*^2^ (2)= 0.502; *P*=.778 |
| Antibiotic drugs *n (%; MD=17)* | 1 (2.9) | 2 (6.7) | 4 (12.1) | *χ*^2^ (2)= 2.213; *P*=.331 |
| Antiarrhythmic drugs *n (%; MD=17)* | 20 (55.6) | 20 (66.7) | 21(65.6) | *χ*^2^ (2)= 1.091; *P*=.580 |
| Glucocorticoids *n (%; MD=17)* | 4 (11.4) | 1 (3.3) | 2 (6.1) | *χ*^2^ (2)= 1.684; *P*=.431 |

^a^ Nonsteroidal anti-inflammatory drug (NSAID).

^b^Anti-depressant, anti-diabetic, anti-biotic, anti-arrhythmic drugs, or glucocorticoids.

**Table S5.** Further Outcome Variables

|  | (A) SMC | (B) SUPPORT | (C) EXPECT | test statistic |
| --- | --- | --- | --- | --- |
| Patients with adverse events after CABG, *n (%)*^b^ | |  |  |  |
| Follow-up | 13 (38.24) | 10 (33.33) | 9 (28.13) | *χ*^2^ (2)= .758; *P*=.684 |
| Patients’ maximum stress electrocardiogram performance in watts^a^ | |  |  |  |
| Follow-up | 115.22 (102.50-127.94) | 130.68 (117.67-143.69) | 125.69 (114.36-137.02) | *F(*2,71)= 1.516; *P*=.227 |
